# Phosphoglycerate Kinase Is Involved in Carbohydrate Utilization, Extracellular Polysaccharide Biosynthesis, and Cell Motility of *Xanthomonas axonopodis* pv. *glycines* Independent of Clp

**DOI:** 10.3389/fmicb.2020.00091

**Published:** 2020-02-07

**Authors:** Wei Guo, Jie Gao, Hong-Jie Wang, Ru-Yi Su, Chu-Yun Sun, Si-Han Gao, Jian-Zhong Liu, Gong-You Chen

**Affiliations:** ^1^Institute of Plant Genetics and Developmental Biology, College of Chemistry and Life Sciences, Zhejiang Normal University, Jinhua, China; ^2^College of Agriculture and Biology, Shanghai Jiao Tong University, Shanghai, China

**Keywords:** *Xanthomonas axonopodis* pv. *glycines*, phosphoglycerate kinase, Crp-like protein, carbohydrate utilization, extracellular polysaccharide, cell motility

## Abstract

Phosphoglycerate kinase (Pgk), catalyzing the reversible conversions between glycerate-1.3-2P and glycerate-3P, plays an important role in carbohydrate metabolism. Here, we show that a Pgk-deficient mutant (NΔ*pgk*) of *Xanthomonas axonopodis* pv. *glycines* (*Xag*) could grow in medium with glucose, galactose, fructose, mannose, or sucrose, as the sole carbon source, suggesting that *Xag* may employ Entner-Doudoroff (ED) and pentose phosphate pathway (PPP), but not glycolysis, to catabolize glucose. NΔ*pgk* could not utilize pyruvate, suggesting that Pgk might be essential for gluconeogenesis. Mutation in *pgk* led to a reduction of extracellular polysaccharide (EPS) biosynthesis, cell motility, and intracellular ATP. As a result, the virulence of NΔ*pgk* was significantly compromised in soybean. NΔ*pgk* could be fully complemented by the wild-type *pgk*, but not by *clp* (encoding Crp-like protein). qRT-PCR analyses demonstrated that *pgk* is regulated by the HrpG/HrpX cascade, but not by Clp. These results suggest that Pgk is involved in carbohydrate utilization, EPS biosynthesis, and cell motility of *Xag* independent of Clp.

## Introduction

*Xanthomonas axonopodis* pv. *glycines* (*Xag*) is widely distributed in soybean-producing areas throughout the world (Chatnaparat et al., 2012). The pathogen enters soybean leaves through stomata or wounds, and reproduces within the intercellular spaces of the spongy mesophyll, resulting in erumpent pustules surrounded by yellow halos (Guo et al., 2019). *Xag* is a quarantine pathogen that can be spread by rain droplets during the growing season and by seed transportation (Liu et al., 2016). Bacterial pustule, caused by *Xag*, is an important bacterial disease on soybean, resulting in premature defoliation and decreasing yield and seed quality (Thowthampitak et al., 2008; Athinuwat et al., 2009).

Over the past two decades, significant progress has been made on elucidating the association of carbon metabolism to virulence and quorum sensing (QS) in *Xanthomonas* spp., such as in *X. oryzae* pv. *oryzicola* (*Xoc*), which infects rice (Guo et al., 2017). Results of these studies have shown that carbohydrate acquisition is essential for the pathogen to grow and establish a successful infection within the hosts (Tang et al., 2005; Mellgren et al., 2009). When the pathogen gains entry into the host plant, it first propagates in the apoplastic space until reaching a density threshold, followed by the expression of lots of virulence-related genes and secretion of cell-wall degrading enzymes through the diffusible signal factor (DSF)-mediated QS mechanism (Büttner and Bonas, 2010). The pathogen overcomes host defenses, adapts to the hostile environment, and ultimately degrades the plant parenchyma cells to gain access to its nutritional reservoirs (Tamir-Ariel et al., 2007; Zhou et al., 2013).

*Xanthomonas* spp. carry out catabolic processes, such as glycolysis, Entner-Doudoroff (ED), pentose phosphate pathway (PPP), and the tricarboxylic acid (TCA) cycle, to catabolize glucose and other simple sugars (sucrose, fructose, mannose, and galactose) (Lu et al., 2009; Kim et al., 2010). These metabolic pathways provide nutrition for growth and propagation of pathogen and supply energy that drives energy-requiring activities such as extracellular polysaccharide (EPS) production and cell motility (Mellgren et al., 2009; Guo et al., 2017). Phosphoglycerate kinase (Pgk), reversibly converting glycerate-1.3-2P to glycerate-3P, is involved in glycolysis, ED, PPP, and gluconeogenesis in *Xag*. However, little is known about the biological processes in which Pgk participates.

Acetyl-CoA and amino acids, produced by catabolic processes, act as precursor molecules for synthesizing DSF signals (Zhou et al., 2015). Crp-like protein (Clp), located at the end of the DSF signaling pathway, is the global regulator in *Xanthomonas* spp. In addition to DSF signals, Clp could also converge on low-oxygen signals transduced by the RavS/RavR two-component regulatory system (He et al., 2009) and environmental signals transduced by the HrpG/HrpX cascade (Wengelnik and Bonas, 1996; Wengelnik et al., 1996). As a consequence, the activated Clp directly or indirectly regulates the expression of downstream genes and modulates numerous biological properties, such as carbohydrate utilization, cell motility, EPS production, and synthesis of extracellular enzymes (Guo et al., 2019), which are all essential for virulence and adaptation of *Xanthomonas* spp. in host plant. However, it is unknown whether Clp could completely or partially complement the impaired properties in the *pgk* mutant. In addition, the inherent relationship between Clp and Pgk is unclear.

In this study, we aimed to explore the biological properties of Pgk and to reveal its association with Clp. Here, we present evidence that Pgk is involved in carbohydrate utilization, cell motility, and EPS biosynthesis independent of Clp in *Xag*. In addition, the expression of *pgk* is regulated by the HrpG/HrpX cascade, but not by DSF signals or Clp.

## Materials and Methods

### Bacterial Strains, Plasmids, and Culture Conditions

Strains and plasmids used in this study are listed in Supplementary Table S1. Unless otherwise specified, *Xag* NEAU001 and its derivative strains were grown at 28°C in NYG (5 g L^–1^ polypeptone, 3 g L^–1^ yeast extract, 20 g L^–1^glycerol), NY (NYG without glycerol), or NCM (2 g L^–1^ (NH_4_)_2_SO_4_, 0.2 g L^–1^ MgSO_4_⋅7H_2_O, 4 g L^–1^ K_2_HPO_4_, 6 g L^–1^ KH_2_PO_4_) media (Liu et al., 2019). *Escherichia coli* strains were routinely grown at 37°C in LB (10 g L^–1^ tryptone, 10 g L^–1^ NaCl, 5 g L^–1^ yeast extract) medium. Antibiotics were added at the following concentrations: ampicillin, 50 μg mL^–1^; kanamycin, 25 μg mL^–1^; spectinomycin, 100 μg mL^–1^; and carbenicillin, 50 μg mL^–1^.

### Generation of the *pgk* Deletion Mutant, Complementation, and By-Path Complementation Strains

The in-frame deletion mutant of *pgk* in *Xag* was generated by using homologs recombination as described by Li et al. (2011), using pKMS1 as a suicide vector and the primers listed in Supplementary Table S2. The obtained mutant was named NΔ*pgk*. The complemented and by-path complemented strains were also constructed by separately introducing the recombinant plasmids pCpgk and pCclp into NΔ*pgk* (Li et al., 2011). The resulting complemented and by-path complemented strains were designated as CNΔ*pgk* and NΔ*pgk*(*clp*), respectively.

### Qualitative/Quantitative Analysis of Carbohydrate Utilization and EPS Production

Carbohydrate utilization and EPS production assays were measured as previously described by Lu et al. (2009). Each experiment was repeated at least in triplicate.

### Determination of Cell Motility and Exoenzyme Activity

Cell motility of *Xag* strains was investigated on plates of semi-solid NY medium with 0.3% agar as described by Long et al. (2018). The exoenzyme activities of *Xag* strains in the supernatants of the cultures were analyzed on NY plates supplemented with different substrates (such as skimmed milk for protease, carboxymethyl cellulose for carboxymethylcellulase, soluble starch for α-amylase, and locust bean gum for endo-β-mannanase) following the method described previously by Thowthampitak et al. (2008). The experiment was repeated independently in triplicate, and five replicate plates were conducted for each treatment.

### Determination of Intracellular ATP

Intracellular ATP levels were determined by using an ATP Assay Kit (Beyotime), following the manufacturer’s protocols. All samples were measured at least in triplicate.

### Sensitivity to Hydrogen Peroxide (H_2_O_2_)

H_2_O_2_ resistance assays were performed as described previously by Wang et al. (2013). Briefly, the fresh *Xag* strains were grown in NY medium at 28°C until the exponential growth phase was reached (OD_600_ ≈ 1.0). Then, the semi-solid NY plates mixed with *Xag* were prepared. After solidification, the saturated filter papers with 0.5 or 1.0 mM H_2_O_2_ were placed on the center of the plates. These bioassay plates were then incubated at 28°C for 2 days. H_2_O_2_ resistance was indicated by measuring the diameter of the zone of inhibition. The same experiment was repeated at least three times.

### qRT-PCR

In addition to *pgk*-related strains, the following strains were used in the qRT-PCR: the deletion mutants of *rpfF* (responsible for the synthesis of DSF), *rpfC*, *rpfG* (the RpfC/RpfG two-component system is involved in sensing and transduction of DSF) (Cai et al., 2017), *rpfB* (required for DSF signals turnover) (Wang et al., 2016), *rpfS* (a second sensor for DSF), *rpfR* (putative interplay with *rpfS*) (An et al., 2014), and *clp* (Guo et al., 2019) in the DSF signaling pathway; the deletion mutants *ravS* and *ravR* (the RavS/RavR two-component system is involved in sensing and transduction of low-oxygen signals) (He et al., 2009); and the deletion mutants of *hrpX*, *hrpG*, and their upstream regulatory genes, such as *rsmA* (Andrade et al., 2014), *zur* (Huang et al., 2009), *trh* (Li et al., 2011), and *xopL* (also called *lrpX*) (Islam et al., 2009), which are all involved in regulating the mRNA level of *clp* in *Xag* (Guo et al., 2019).

All tested strains were incubated in NY medium until OD_600_ ≈ 2.0. Total RNA was extracted using the Trizol reagent (Invitrogen), following the manufacturer’s protocol. cDNA synthesis was conducted with a *PrimeScript*^TM^ RT reagent Kit (TaKaRa). The transcriptional levels of tested genes were determined by qRT-PCR using the primers listed in Supplementary Table S2. qRT-PCR was performed on the Applied Biosystems^TM^ 7500 Real-Time PCR System using SYBR *Premix ExTaq*^TM^ (TaKaRa). The transcriptional level of integration host factor A (*ihfA*) was used as a reference (Thowthampitak et al., 2008). All qRT-PCR analyses were performed in two independent experiments.

### Plant Assays

The virulence of *Xag* strains was assessed as described previously (Chatnaparat et al., 2012). Briefly, all tested strains were grown in NY medium with shaking until the exponential phase was reached. Bacterial cells were then harvested, washed twice, and re-suspended to OD_600_ ≈ 0.2. Then bacterial cells were high-pressure sprayed into soybean leaves (*Glycine max* cv. Zhoudou63; susceptible to bacterial pustule, 6 weeks old). Phenotypes were scored 15 days post-inoculation. The experiments were repeated at least in triplicate.

Bacterial cell suspensions at OD_600_ ≈ 0.1 were infiltrated into the intercellular spaces of leaves with needleless syringes. Bacterial growth within soybean leaves was assessed as previously described by Liu et al. (2014). The experiments were repeated at least in triplicate.

### Statistical Analysis

Means and standard deviations (SD) of experimental data were calculated using Microsoft Office Excel. Statistical analyses were performed using a Student’s *t*-test. ^∗^Indicates significance at *P* < 0.05; ^∗∗^indicate significance at *P* < 0.01.

## Results

### Deletion Mutant of *pgk* From *X. axonopodis* pv. *glycines*

**FIGURE 1:**
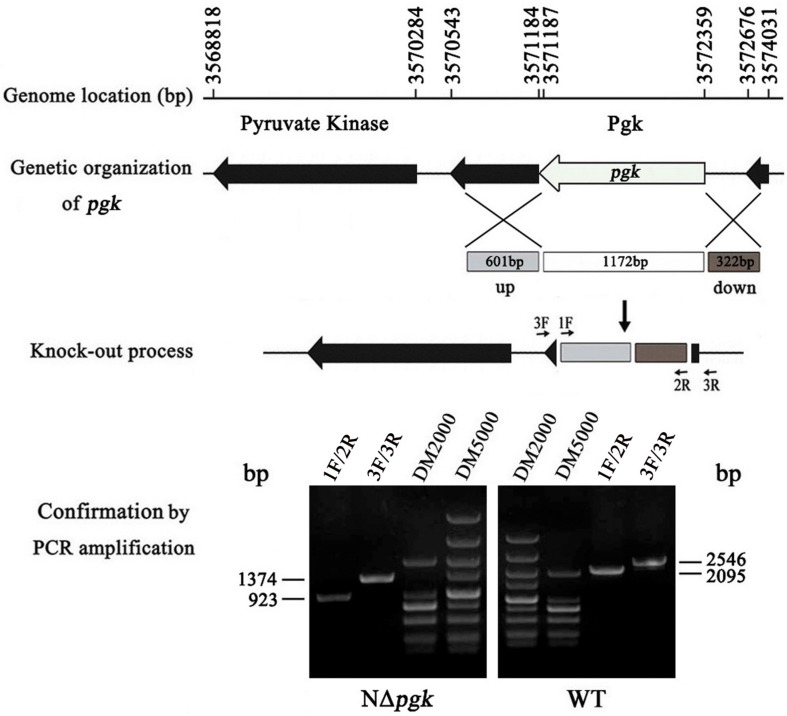
Schematic map and molecular analysis of *pgk* mutation in *X. axonopodis* pv. *glycines* (*Xag*). The positions and orientations of *pgk*, encoding phosphoglycerate kinase (Pgk), and other adjacent open reading frames (ORFs) are shown using the genome sequence of *Xag* 12-2 strain as a reference. Numbers and arrows respectively represent the locations and orientations of the ORFs; lines indicate intergenic sequences. A non-polar construction of the *pgk* deletion mutant was sketched. The gray and brown box indicated where the left and right flanks targeted *pgk*. The white box shows the 1,172 bp deletion of *pgk*. *pgk* was knocked out after two homologous crossover events occurred, and then was verified by nested PCR with two primer pairs 1F/2R and 3F/3R.

Genomic sequence analysis showed that only one open reading frame, in the genome of *Xag* strain NEAU001, is annotated to encode Pgk. To facilitate the functional study of *pgk*, a non-polar deletion mutant was constructed by using homologous recombination and pKMS1 as a suicide vector. As expected, a 2,095 bp fragment was amplified from the wild-type *Xag* strain with the primer pairs 1F/2R, whereas only a 923 bp fragment was amplified in the *pgk* mutant because of an in-frame deletion of 1,172 bp from *pgk* (Figure 1). The in-frame deletion was further verified by a nested PCR using the primer pairs 3F/3R (Figure 1). The deletion mutant, designated NΔ*pgk*, was used in our studies.

### Pgk Is Involved in the Utilization of Carbohydrates in *Xag* Independent of Clp

Phosphoglycerate kinase is an indispensable component of glycolysis, ED, PPP, and gluconeogenesis (Kim et al., 2010); therefore, we measured the growth of NΔ*pgk* to determine whether Pgk is involved in carbohydrate metabolism in *Xag*. We found that NΔ*pgk* has a similar growth pattern to that of the wild-type *Xag* when grown in NY medium (Supplementary Figure S1), indicating that NΔ*pgk* was not auxotrophic. To qualitatively assess the effect of Pgk on carbohydrate utilization, we used NCM medium, which is similar to the conditions encountered in the plant apoplast, to investigate the growth of NΔ*pgk*. When glucose, galactose, fructose, mannose, sucrose, or pyruvate was used as the sole carbon source, the *pgk* mutant displayed smaller colonies than the wild-type strain (Supplementary Figure S2), suggesting that mutation in *pgk* diminishes the ability of *Xag* to utilize carbohydrates.

**FIGURE 2:**
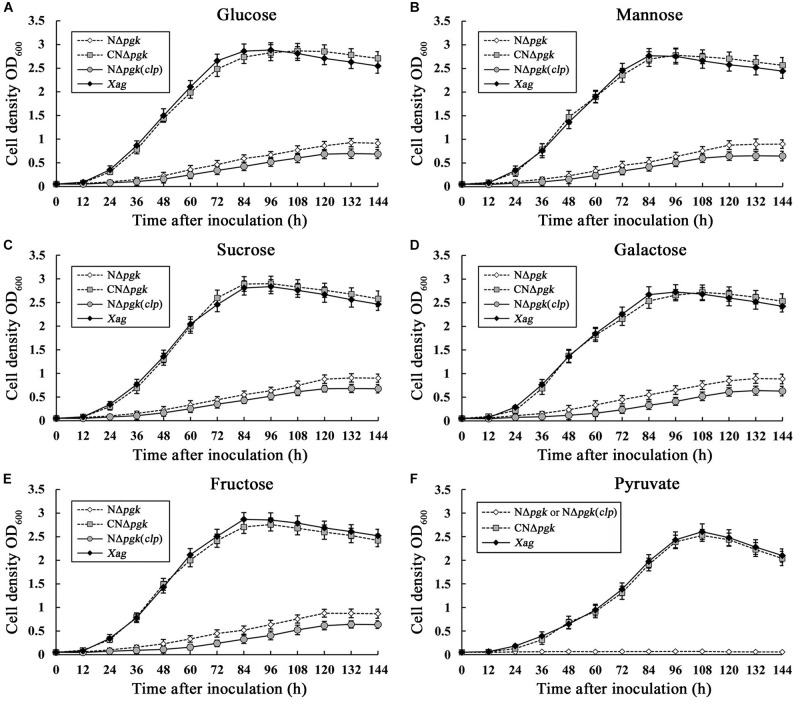
Phosphoglycerate kinase is involved in the utilization of glucose **(A)**, mannose **(B)**, sucrose **(C)**, galactose **(D)**, fructose **(E)**, and pyruvate **(F)** in *Xag* independent of Clp. Aliquots were taken in triplicate at intervals of 144 h after incubation at 28°C, and bacterial growth was determined by measuring OD_600_ against the medium blank. Data are means ± standard deviations (SD) from three repeats.

To quantitatively evaluate the contribution of Pgk to carbohydrate utilization, the growth of NΔ*pgk*, the complemented strain CNΔ*pgk*, the by-path complemented strain NΔ*pgk*(*clp*), and the wild-type strain was tested in NCM liquid medium supplemented with different carbon sources. When glucose, galactose, fructose, mannose, or sucrose was used as the sole carbon source, NΔ*pgk* exhibited significantly slower growth compared to the wild-type strain (*P* < 0.01), and N*Δpgk* harboring the *pgk in trans* completely restored the ability to acquire these sugars (Figure 2). Compared with the growth of NΔ*pgk*, the growth rate of NΔ*pgk*(*clp*) was lower (Figure 2), indicating a reduced ability to acquire sugars supplemented and an optimal level of Clp is required for *Xag* to utilize carbohydrates. When pyruvate was used as the sole carbon source, the growths of both NΔ*pgk* and NΔ*pgk*(*clp*) were similarly limited (Figure 2). These results revealed that Pgk is required for *Xag* to utilize glucose, galactose, fructose, mannose, sucrose, and especially pyruvate.

**FIGURE 3:**
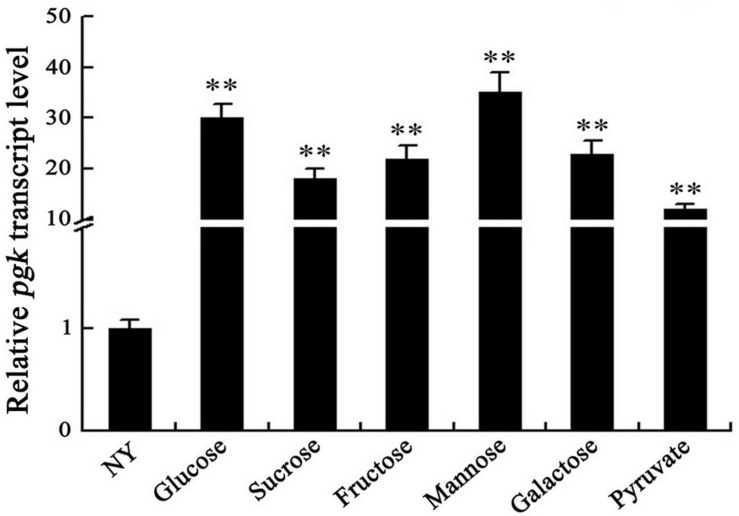
Carbohydrates induce the expression of *pgk* in *Xag*. *ihfA* was used as an internal control for data analyses. Data are means ± SD from three replicates. The asterisks in each horizontal column indicate significant differences at *P* < 0.01.

The expression of *pgk* was assessed using qRT-PCR after the wild-type was grown in NY medium supplemented with glucose, galactose, fructose, mannose, sucrose, or pyruvate. We found that *pgk* was strongly induced by these carbohydrates, with a minimum of 10 times higher than that in NY medium not supplemented with any sugar (Figure 3). These results indicate that the expression of *pgk* was significantly enhanced by carbohydrates involved in the carbon metabolic pathways, further supporting that Pgk plays a crucial role in carbohydrate metabolism.

### Pgk Is Involved in EPS Biosynthesis of *Xag* Independent of Clp

**FIGURE 4:**
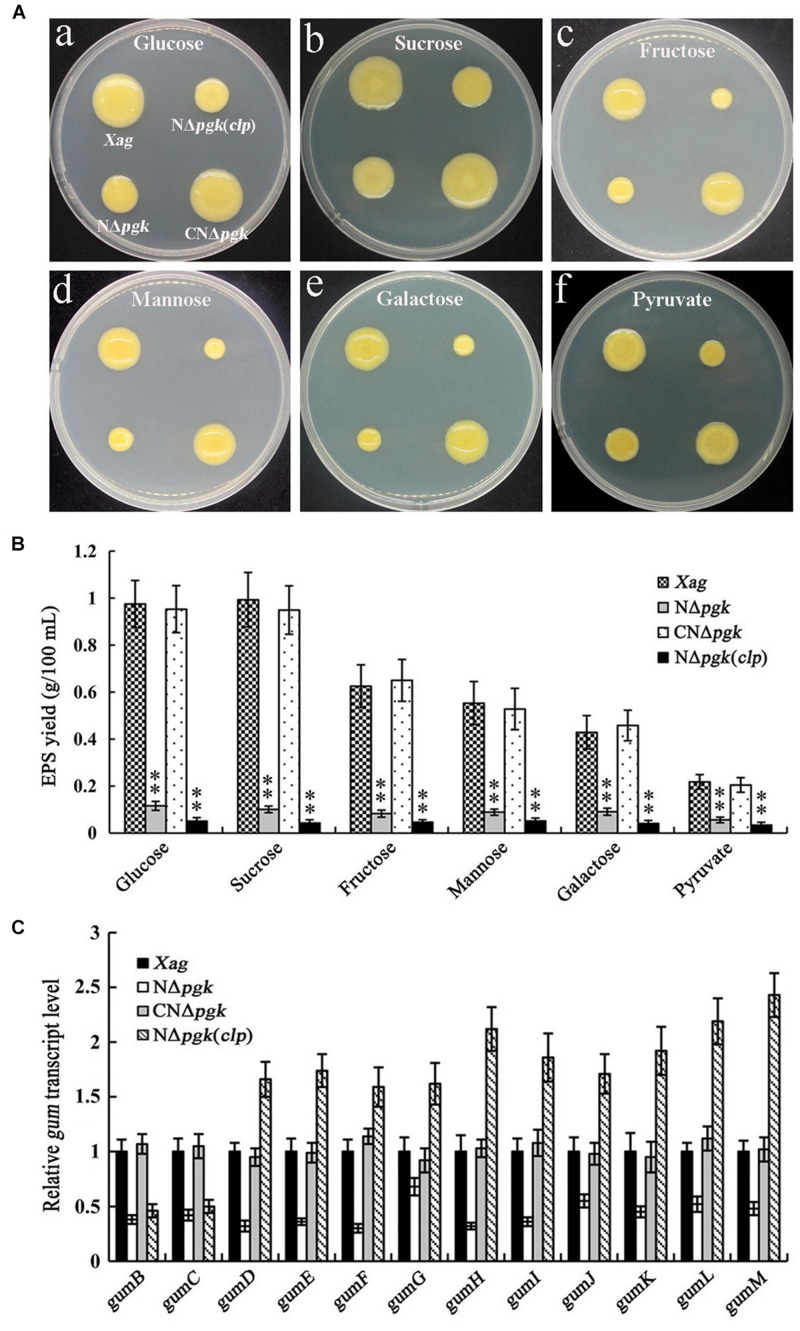
Phosphoglycerate kinase is involved in the biosynthesis of EPS in *Xag* independent of Clp. EPS productions of the *pgk* mutant (NΔ*pgk*), the NΔ*pgk* complemented strain (CNΔ*pgk*), and the by-path complemented strain [NΔ*pgk*(*clp*)] in NY medium supplemented with 2% glucose, galactose, fructose, mannose, sucrose, or pyruvate were determined by qualitative **(A)** and quantitative **(B)** methods. **(C)** The transcriptional levels of *gum* genes in NΔ*pgk*, CNΔ*pgk*, and NΔ*pgk*(*clp*) were detected by qRT-PCR. Data are means ± SD from three repeats. The asterisks in each horizontal column indicate significant differences at *P* < 0.01.

Because Pgk is essential for glycolysis, ED, PPP, and gluconeogenesis (Kim et al., 2010), we further explored whether a mutation in *pgk* has any effect on EPS biosynthesis in *Xag*. All tested strains were grown on NY plates supplemented with 2% glucose, galactose, fructose, mannose, sucrose, or pyruvate. NΔ*pgk* had smaller colonies than the wild-type strain on all plates tested (Figure 4A), indicating that Pgk might be involved in the biosynthesis of EPS in *Xag*.

The contribution of Pgk to EPS production was quantitatively examined by culturing all strains in NY liquid medium supplemented with 2% carbohydrates for 5 days. NΔ*pgk* produced approximately 35–85% less EPS than the wild-type when cultured in glucose-, galactose-, fructose-, mannose-, sucrose-, or pyruvate-containing medium (Figure 4B). In addition, the EPS yield of CNΔ*pgk* was fully restored to the wild-type level (Figure 4B). These results demonstrate that Pgk is involved in the biosynthesis of EPS in *Xag*.

We next determined whether Clp could, either completely or partially, restore EPS production in NΔ*pgk*. Our results showed that by-path complementation of *clp* in NΔ*pgk* did not restore EPS biosynthesis, on the contrary, it showed weakened EPS biosynthesis (Figures 4A,B). To further understand how Pgk is involved in the biosynthesis of EPS in *Xag*, qRT-PCRs were performed to explore the transcriptional levels of *gum* and *xan* genes. As shown in Figure 4C, the expression of 12 *gum* genes in NΔ*pgk* was significantly lower than in CNΔ*pgk* and the wild-type strain, indicating that the mutation in *pgk* negatively regulates the expression of *gum* genes. The transcriptional levels of *gumB* and *gumC* in NΔ*pgk*(*clp*) was significantly lower than that in the wild-type strain, while the transcriptional levels of *gumD-M* in NΔ*pgk*(*clp*) was significantly higher than that in the wild-type strain, suggesting that by-path complementation of *clp* up-regulates the expression of *gum* genes except for *gumB* and *gumC*, which play a critical role in the biosynthesis of EPS in *Xag*. However, a minimal change in the expression level of *xan* genes was observed in NΔ*pgk* or NΔ*pgk*(*clp*) (Supplementary Figure S3). These results demonstrate that Pgk is involved in the biosynthesis of EPS in *Xag* independent of Clp, possibly through partially inhibiting the expression of the *gum* gene cluster.

### Pgk Is Involved in Cell Motility of *Xag* Independent of Clp

**FIGURE 5:**
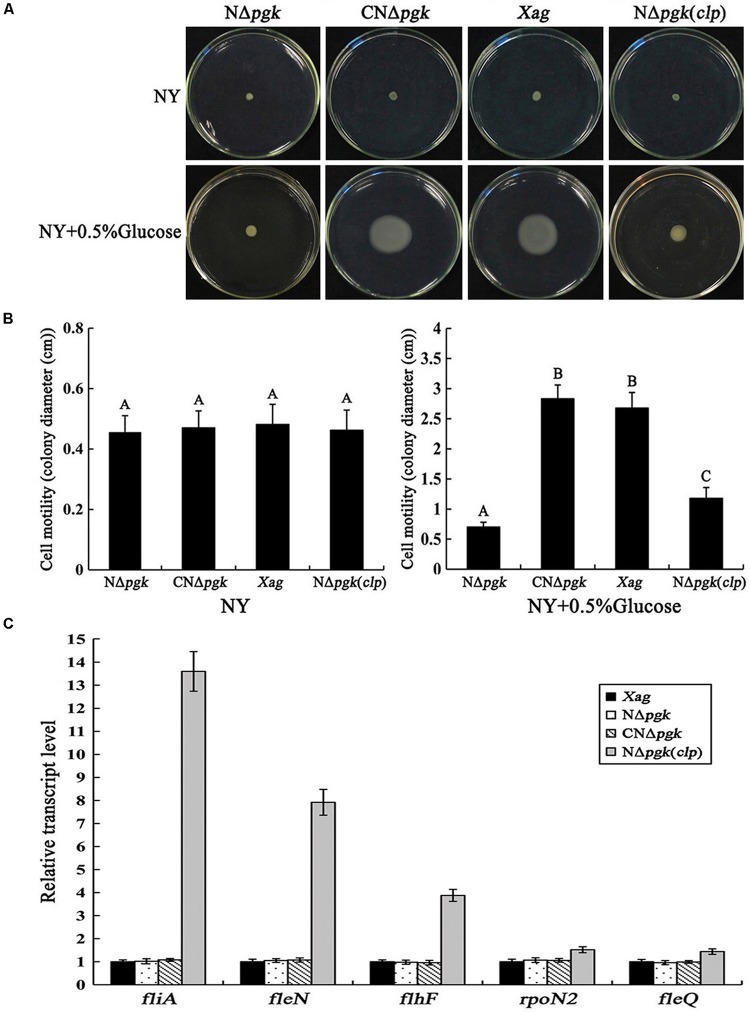
Phosphoglycerate kinase is involved in cell motility of *Xag* independent of Clp. **(A)** Cell motility of NΔ*pgk*, CNΔ*pgk*, and NΔ*pgk*(*clp*) was detected on NY plates or NY plates supplemented with 0.5% glucose and photographed after 1–2 days of incubation at 28°C. **(B)** Swimming diameter of NΔ*pgk*, CNΔ*pgk*, and NΔ*pgk*(*clp*) on plates was measured. **(C)** The transcriptional levels of the flagellar-associated genes in NΔ*pgk*, CNΔ*pgk*, and NΔ*pgk(clp)* were examined by qRT-PCR. Data are means ± SD from three replicates. The different letters above the horizontal columns represent significant differences at *P* < 0.01.

We explored whether a mutation in *pgk* affects the cell motility of *Xag*. Our results showed that there was little visible difference in cell motility among NΔ*pgk*, CNΔ*pgk*, NΔ*pgk*(*clp*), and the wild-type strain on NY plates (Figure 5A). However, on NY plates supplemented with 0.5% glucose, N*Δpgk* displayed severely reduced cell motility compared with the wild-type strain (Figure 5A). The diameter of the colonies resulting from migration away from the inoculation sites on the surface of the plate was approximately 0.72 cm for N*Δpgk*, 2.83 cm for CN*Δpgk*, 1.21 cm for NΔ*pgk*(*clp*), and 2.74 cm for the wild-type strain (Figure 5B). A *t-*test indicated that the mean diameter of N*Δpgk* was significantly smaller than that of the wild-type strain (*P* < 0.01), and the cell motility of CNΔ*pgk* was fully restored to the wild-type level.

We then investigated whether Clp could, either partially or completely, restore cell motility of NΔ*pgk*. Interestingly, by-path complementation of *clp* in NΔ*pgk* only slightly enhanced the cell motility of NΔ*pgk* compared to that of the wild-type (Figure 5B). To further understand how Pgk is involved in cell motility, qRT-PCRs were performed to explore the expression of *fliA*, *fleN*, *flhF*, *rpoN2*, and *fleQ*, which have been shown crucial for cell motility (Guo et al., 2019). As shown in Figure 5C, no change in the expression of these five genes was observed in NΔ*pgk*, CNΔ*pgk*, or the wild-type strain. However, the transcription of these five genes, especially *fliA* and *fleN*, was significantly increased in NΔ*pgk*(*clp*) (*P* < 0.01) (Figure 5C). As reported previously, the overexpression of *clp* in the wild-type background also results in the increased expressions of these five genes and slightly enhanced cell motility compared to the wild-type (Guo et al., 2019). Therefore, our results suggest that the by-path complementation of *clp* could promote cell motility, rather than restore the impaired motility of NΔ*pgk*. Further, Pgk is involved in the cell motility of *Xag* independent of Clp.

### Pgk Is Required for Producing Intracellular ATP in *Xag*

**FIGURE 6:**
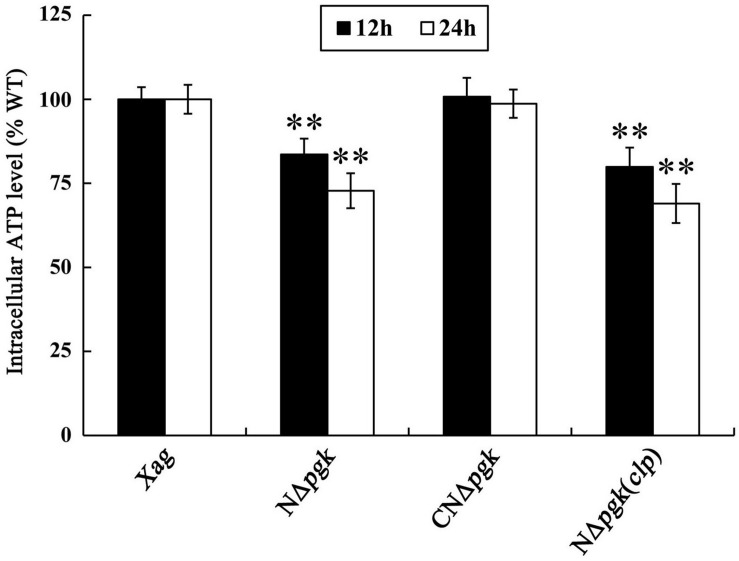
Phosphoglycerate kinase is required for producing intracellular ATP of *Xag*. Data are means ± SD from three replicates. The asterisks in each horizontal column indicate significant differences at *P* < 0.01.

Phosphoglycerate kinase is involved in energy-requiring activities, such as EPS biosynthesis and cell motility; therefore, we determine whether Pgk affects intracellular energy production in *Xag*. Our results showed that intracellular ATP of NΔ*pgk* was 83.6 and 72.8% of the wild-type level when cultured for 12 and 24 h, respectively, in NY medium supplemented with 2% glucose (Figure 6). The ATP levels in CN*Δpgk* were completely restored to the wild-type level at all incubation times, indicating that *pgk* is required for producing intracellular ATP in *Xag*. Interestingly, by-path complementation of *clp* in NΔ*pgk* did not increase or maintain intracellular ATP levels of NΔ*pgk*, instead, it slightly decreased the ATP levels (Figure 6). Overall, these results reveal that Pgk is required for the production of ATP in *Xag*.

### *pgk* Is Regulated by the HrpG/HrpX Cascade, but Not by DSF Signals or the Global Regulator Clp in *Xag*

**FIGURE 7:**
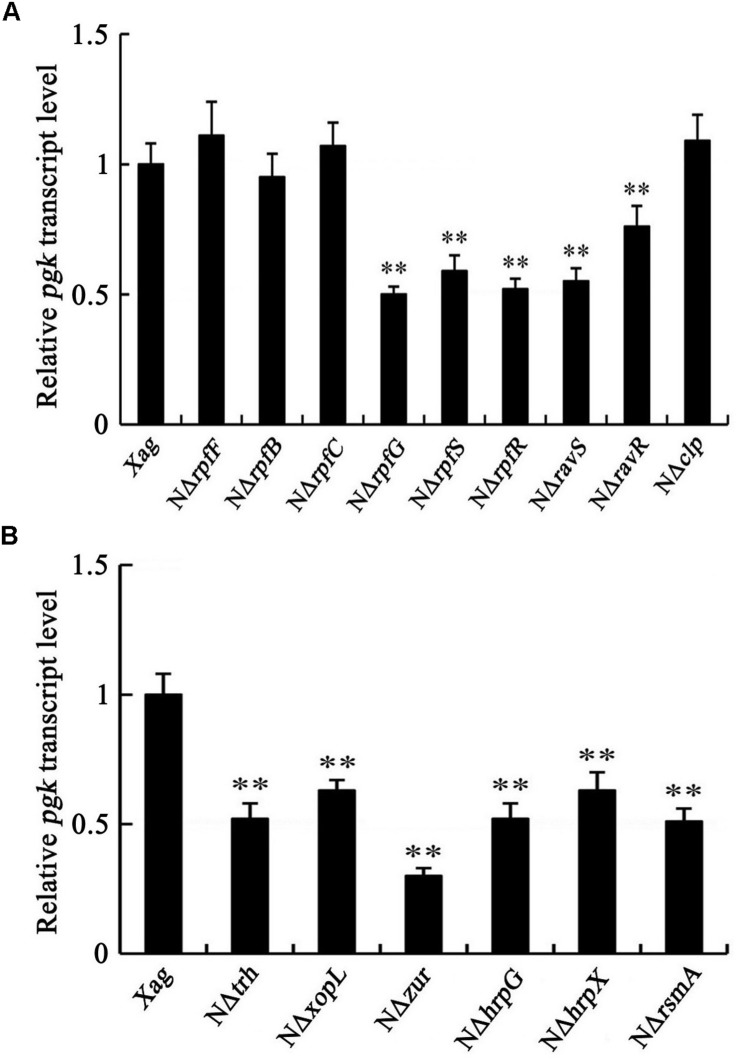
*pgk* is positively regulated by the HrpG/HrpX cascade **(B)**, but not by DSF signals or Clp **(A)**. Data are means ± SD from three replicates. The asterisks in each horizontal column indicate significant differences at *P* < 0.01.

We determined whether the mRNA level of *pgk* is regulated by the DSF signals, low-oxygen signals, and the HrpG/HrpX cascade in *Xag*. Our results showed that the expression of *pgk* in the deletion mutants of *rpfG*, *rpfS*, *rpfR*, *ravS*, and *ravR* was significantly lower than that in the wild-type, but the deletion mutants of *rpfF*, *rpfB*, *rpfC*, and *clp* had similar expression levels as the wild-type strain, implying that the expression of *pgk* is not regulated by DSF signals or the global regulator Clp in *Xag* (Figure 7A). Compared with the wild-type, the expression of *pgk* was reduced in the deletion mutants of *hrpX*, *hrpG*, *trh*, *xopL*, *zur*, and *rsmA*, indicating that *pgk* is positively regulated by the HrpG/HrpX cascade (Figure 7B).

### Pgk Is Not Involved in the Production of Exoenzymes and in H_2_O_2_ Resistance of *Xag*

Because *pgk* was positively regulated by Zur and RsmA, which are involved in regulating detoxification and exoenzymes production in *Xanthomonas* spp. (Büttner and Bonas, 2010), we examined the effect of Pgk in the exoenzymes production and in H_2_O_2_ resistance in *Xag*. We found that the deletion of *pgk* in *Xag* did not affect the production of exoenzymes, such as protease, α-amylase, carboxymethylcellulase, and endo-β-mannanase (Supplementary Figure S4). We have previously demonstrated that either deletion or over-expression of *clp* reduces the protease activity, and Clp positively regulates synthesis of α-amylase, endo-β-mannanase, and carboxymethylcellulase in *Xag* (Guo et al., 2019). Therefore, the halos produced by NΔ*pgk* with by-path complementation *clp* looks smaller in protease assay and larger in α-amylase, carboxymethylcellulase, and endo-β-mannanase assays when compared with NΔ*pgk* (Supplementary Figure S4). In addition, the deletion of *pgk* in *Xag* did not render more sensitive to H_2_O_2_ (Supplementary Figure S5). These results indicate that Pgk is not involved in the production of exoenzymes or in H_2_O_2_ resistance in *Xag*.

### Pgk Contributes to *Xag* Virulence and Growth in Host Soybean

**FIGURE 8:**
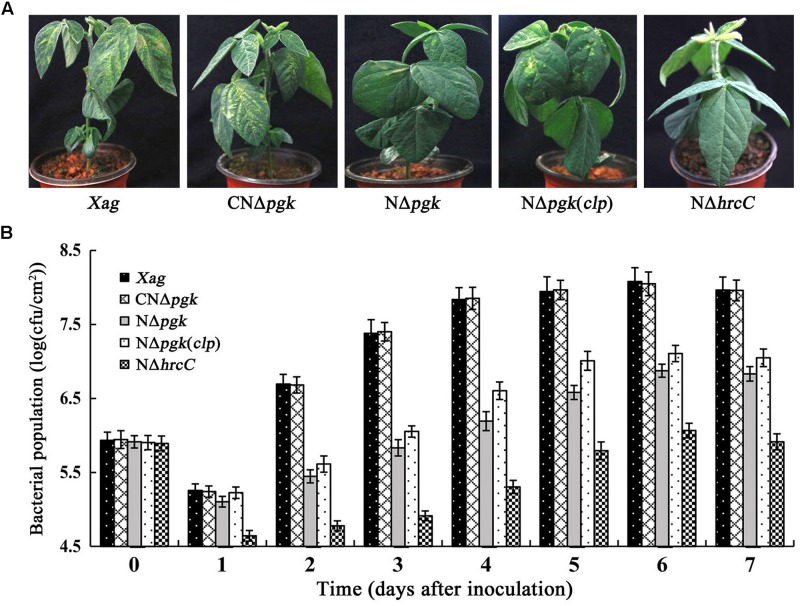
Phosphoglycerate kinase is essential for the growth and virulence of *Xag* in soybean. **(A)** Bacterial pustules caused by *Xag* strains on inoculated leaves of soybean seedlings. Photographs were taken 15 days post-inoculation. **(B)** Bacterial growth in inoculated leaves of soybean. Bacteria were recovered from the inoculated leaves every day for 7 days post-inoculation and homogenized in sterile water. The homogenates were diluted and plated on NY plates with appropriate antibiotics. Bacterial CFU was counted after incubation at 28°C for 3 days. Data are means ± SD from three repeats.

We determined whether Pgk plays a vital role in the virulence of *Xag* in soybean. Our findings indicated that NΔ*pgk* exhibited significantly reduced virulence in soybean relative to the wild-type (Figure 8A), and CNΔ*pgk* had similar virulence as the wild-type. Furthermore, NΔ*pgk*(*clp*) showed increased virulence compared with NΔ*pgk*, but less virulence compared with CNΔ*pgk*. The T3SS deletion mutant NΔ*hrcC* completely lost virulence on soybean (Figure 8A).

To determine whether the impaired virulence of NΔ*pgk* is associated with decreased bacterial growth, we explored the growth of bacterial cells infiltrated into soybean leaves. The colonies of NΔ*pgk* were significantly fewer than that of the wild-type strain at each of time points (*P* < 0.01). The growth of NΔ*pgk* could be fully restored by *pgk in trans*, whereas NΔ*hrcC* did not show increased growth in inoculated soybean tissue (Figure 8B). In addition, NΔ*pgk*(*clp*) showed increased growth in soybean leaves compared with NΔ*pgk*, but was less than that of CNΔ*pgk* (Figure 8B). Taken together, these results indicate that *pgk* is required for virulence and growth of *Xag*, and by-path complementation of *clp* is beneficial to the virulence of NΔ*pgk* in host soybean.

## Discussion

In this study, we found that Pgk plays an important role in the catabolism of glucose and other simple sugars in *Xag*. NΔ*pgk* showed impaired growth when glucose, galactose, fructose, mannose, or sucrose was the sole carbon source (Figure 2). However, NΔ*pgk* still showed certain level of growth in medium with these individual sugars as the sole carbon source, suggesting that *Xag* may employ ED and PPP, but not glycolysis, to utilize these sugars. This is consistent with the findings from previous studies conducted in the parenchyma pathogen *Xoc* (Guo et al., 2015), as well as the vascular pathogens *X. campestris* pv. *campestris* (*Xcc*) (Lu et al., 2009) and *X. oryzae* pv. *oryzae* (*Xoo*) (Kim et al., 2010), which lack phosphofructokinase activity required for glycolysis. In addition, NΔ*pgk* shows no growth in medium with pyruvate as the sole carbon source (Figure 2), implying that inactivation of Pgk probably results in a severing of the gluconeogenic pathway. Similarly, this has also been observed in *Xoc* (Guo et al., 2017) and *Xcc* (Tang et al., 2005), in which the functional gluconeogenic pathway is necessary for the acquisition of pyruvate. Therefore, we deduce that *Xag*, probably similar to *Xoc* and *Xcc*, possesses an identical carbon metabolic pathway, using ED in conjunction with TCA to catabolize glucose. Interestingly, introduction of the recombinant plasmid pCclp into NΔ*pgk* results in further impairment of the utilization of carbohydrates. This is consistent with results from a previous study on the overexpression of *clp* in *Xag*, which results in reduced utilizations of carbohydrates (Guo et al., 2019). Therefore, we speculate that overexpression or by-path complementation of *clp* may directly or indirectly inhibit the activity of a key metabolic enzyme from carbon metabolic pathways, leading to the limited acquisition of carbohydrates in *Xag*.

Our data showed that deletion of *pgk* in *Xag* resulted in significantly reduced EPS production (Figures 4A,B). This was further supported by the qRT-PCR results that showed that the expression of *gum* genes was significantly reduced in NΔ*pgk* (Figure 4C). We also found that a mutation in *pgk* in *Xag* resulted in significantly reduced cell motility (Figure 5), which is similar to *pgi* (encoding glucose-6-phosphate isomerase) and *zwf* (encoding glucose-6-phosphate dehydrogenase) of *Xoc* (Guo et al., 2015, 2017). However, the decreased motility was not correlated with the expression of *fliA*, *flhF*, *rpoN2*, *fleN*, and *fleQ* (Figure 5C), which are required for cell motility (Tian et al., 2015). Pgk is involved in the reversible conversions between glycerate-1.3-2P and glycerate-3P in carbon metabolic pathways. Thus it is indicated that Pgk has a dual function in energy generation or consumption. Pgk is the producer of ATP in glycolytic pathway, but it is an ATP consumer in gluconeogenic pathway. In order to explore the possible reasons for the decrease of EPS synthesis and cell motility in NΔ*pgk*, we investigated the intracellular ATP level, which provides energy to drive these activities (Chao et al., 2008). We found that mutation in *pgk* led to a reduced intracellular ATP level (Figure 6), which may result from the reduced acquisition of carbohydrates. Thus, we speculate that the reduced intracellular ATP level in NΔ*pgk* may be one of the major causes that negatively affect EPS production and cell motility in this mutant strain.

Previous studies have shown that Clp directly or indirectly regulates diverse biological processes, including EPS production, cell motility, the TCA cycle, and the synthesis of extracellular enzymes (He et al., 2007; Guan et al., 2009). Recently, we also found that Clp functions as both an activator and/or a repressor in multiple biological processes, such as EPS production, cell motility, carbohydrate utilization, and extracellular enzyme activities (Guo et al., 2019). Thus, we further investigated the association between Pgk and Clp in these biological processes. However, by-path complementation of *clp* did not restore of the impaired properties caused by NΔ*pgk*. Although *pgk* contains a putative Clp binding motif AGGCA-N6-TCACA in the promoter region (He et al., 2007), the transcriptional levels of *pgk* in the mutants of *rpfF*, and *clp* are equivalent to that in the wild-type (Figure 7A), indicating that *pgk* is not regulated by DSF signals/Clp regulon in *Xag*. Thus, we speculate that Pgk and Clp likely participate in diverse biological processes, such as carbohydrate utilization, cell motility, and EPS production, via different pathways.

A previous study has shown that the genes encoding catalytic enzymes in carbohydrate metabolic pathways could be regulated by the Hrp system in *Xoc* (Guo et al., 2017). Here, we found that *pgk* is also positively regulated by the HrpG/HrpX cascade and its upstream regulators RsmA and Zur (Figure 7B). Previous studies have reported that RsmA regulated EPS biosynthesis and cell motility, and Zur regulated EPS biosynthesis in *Xanthomonas* spp. (Chao et al., 2008; Huang et al., 2009; Zhu et al., 2011). Further studies should be conducted to investigate whether RsmA and Zur regulate EPS biosynthesis and cell motility partially through Pgk, or if both RsmA and Zur share the same signaling pathway as Pgk.

Our results showed that Pgk is essential for maintaining both virulence and growth of *Xag* in soybean (Figure 8). Attenuation in the virulence and growth of NΔ*pgk* in host plant may have resulted, at least partially, from the combined effects of the compromised utilization of carbohydrates, the impaired production of EPS, and limited cell motility (Huang et al., 2009; Lu et al., 2009; Guo et al., 2019). Nutrient utilization is indispensable for pathogen to grow and colonize within host cells (Mellgren et al., 2009). Therefore, the reduced ability of NΔ*pgk* to utilize carbohydrates in soybean reduces its growth and aggressiveness. In addition, previous studies have suggested that carbohydrates could induce metabolic changes, the secretion of extracellular enzymes, and the expression of virulence-related genes (Watt et al., 2009), which are crucial for the virulence of *Xanthomonas* spp. EPS can enhance susceptibility of host by repressing defense responses such as callose deposition (Yun et al., 2006), mask bacterium to prevent recognition by host (Lu et al., 2009), and contribute to biofilm formation (Dow et al., 2003), which play an important role during pathogen infection in *Xanthomonas* spp. Cell motility allows bacteria to obtain sufficiently nutritional sources, avoid unfavorable environments and disperse effectively, and seems to be required for the pathogenicity of the parenchyma pathogen *Xanthomonas* spp. (Malamud et al., 2011). Interestingly, by-path complementation of *clp* contributes to the virulence of NΔ*pgk* to certain extent. The most likely reason is that significantly increased extracellular enzyme activity probably promotes the infection and colonization of pathogen in host soybean. Plant cell walls act as the first barrier of defense against bacterial invasion. Cell wall-degrading enzymes may facilitate pathogen invasion into host cells by digesting cell walls, thus contribute to disease development (Tayi et al., 2016).

**FIGURE 9:**
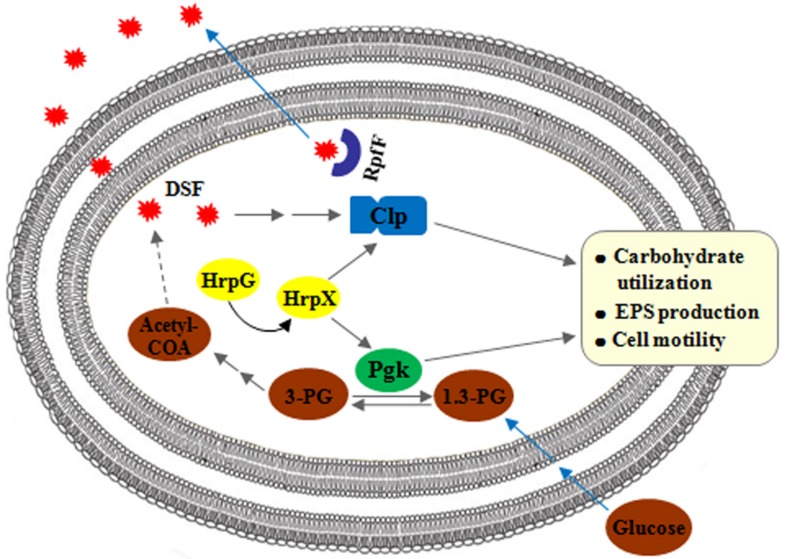
Working model to depict the association between Pgk and Clp in biological processes in *Xag*. Both *pgk* and *clp*, positively regulated by the HrpG/HrpX cascade, are involved in carbohydrate utilization, EPS biosynthesis, and cell motility of *Xag*. However, *pgk* is not regulated by either DSF signals or Clp, and is involved in the above biological properties independent of Clp.

The results from this study, together with the results from our previous study (Guo et al., 2019), advance our understanding of the biological properties of Pgk, an enzyme which reversibly catalyzes the conversions between glycerate-1.3-2P and glycerate-3P, and enable us to propose a working model to depict the functional roles of Pgk and Clp, and understand how their gene expression is regulated in *Xag*. In this model (Figure 9), both *pgk* and *clp* are positively regulated by the HrpG/HrpX cascade, which transduces environmental signals. In addition, *clp* is also positively regulated by DSF signals. However, *pgk* is not regulated by either DSF signals or Clp. Even though Clp and Pgk contribute positively to carbohydrate utilization, EPS biosynthesis, and cell motility, they are not functionally interconnected. It is likely that Pgk contributes to carbohydrate utilization, EPS biosynthesis, and cell mobility through generating metabolic carbon products and/or altering the intracellular ATP levels either directly or indirectly by its enzymatic activities. It seems that the function of Pgk in these processes is totally independent of Clp, the global regulator in *Xanthomonas* spp.

## Data Availability Statement

All datasets generated for this study are included in the article/Supplementary Material.

## Author Contributions

WG, G-YC, and J-ZL conceived the study. WG, JG, H-JW, R-YS, C-YS, and S-HG performed the experiments. WG and J-ZL analyzed the data. WG wrote the manuscript. J-ZL and G-YC revised the manuscript.

## Conflict of Interest

The authors declare that the research was conducted in the absence of any commercial or financial relationships that could be construed as a potential conflict of interest.
