# Supplementary material for: Phosphoglycerate Kinase Is Involved in Carbohydrate Utilization, Extracellular Polysaccharide Biosynthesis, and Cell Motility of Xanthomonas axonopodis pv. glycines Independent of Clp
Source: Front Microbiol. 2020 Feb 7;11:91. doi: 10.3389/fmicb.2020.00091 (PMC7018688; doi:10.3389/fmicb.2020.00091)

**SUPPLEMENTARY MATERIAL**

**TABLE S1 |** Strains and plasmids used in this study.

**TABLE S2 |** Primers used in this study.

**FIGURE S1 |** Growth curve of *Xag* strains on NY liquid medium. Data are means ± SD from three repeats.

**FIGURE S2 |** Determination of growth capacity by *Xag* strains on NCM plates supplemented with 0.5% glucose, galactose, fructose, mannose, sucrose, or pyruvate as the sole carbon source. Photographs were taken 5 days post-inoculation at 28°C.

**FIGURE S3 |** The transcriptional levels of *xan* genes in NY liquid medium.

**FIGURE S4 |** Pgk is not involved in the production of exoenzymes by *Xag*. Filtrates from bacterial cultures were placed in wells in assay media to assess protease (a), a-amylase (b), carboxymethylcellulase (c), and endo-β-mannanase (d). Labels in b, c, and d are as shown in figure a. Assays were conducted three times, each with four replicates, and a representative plate for each assay is shown.

**FIGURE S5 |** Pgk is not involved in H_2_O_2_ resistance of *Xag*.

**TABLE S1 |** Strains and plasmids used in this study

| **Strains or plasmids** | | **Relevant characteristics** | **Reference or source** |
| --- | --- | --- | --- |
| *E. coli* | |  |  |
| DH5α | | F^—^ Φ80d*lacZ* ΔM15Δ(*lacZYA-argF*)U169 *endA1 deoR recA1 hsdR17*(r_K_^—^ m_K_^+^) *phoA supE44 λ^—^ thi-l gyrA96 relA1* | Clontech |
| *X.* *axonopodis* pv. *glycines* |  | |  |
| NEAU001 | | Wild-type, the causal agent of bacterial pustule in soybean, Cb^r^ | This lab |
| N∆*clp* | | *clp* deletion mutant of strain NEAU001, Cb^r^ | This lab |
| N∆*hrcC* | | *hrcC* deletion mutant of strain NEAU001, Cb^r^ | This lab |
| N∆*rpfF* | | *rpfF* deletion mutant of strain NEAU001, Cb^r^ | This lab |
| N∆*rpfB* | | *rpfB* deletion mutant of strain NEAU001, Cb^r^ | This lab |
| N∆*rpfC* | | *rpfC* deletion mutant of strain NEAU001, Cb^r^ | This lab |
| N∆*rpfG* | | *rpfG* deletion mutant of strain NEAU001, Cb^r^ | This lab |
| N∆*rpfS* | | *rpfS* deletion mutant of strain NEAU001, Cb^r^ | This lab |
| N∆*rpfR* | | *rpfR* deletion mutant of strain NEAU001, Cb^r^ | This lab |
| N∆*ravS* | | *ravS* deletion mutant of strain NEAU001, Cb^r^ | This lab |
| N∆*ravR* | | *ravR* deletion mutant of strain NEAU001, Cb^r^ | This lab |
| N∆*trh* | | *trh* deletion mutant of strain NEAU001, Cb^r^ | This lab |
| N∆*xopL* | | *xopL* deletion mutant of strain NEAU001, Cb^r^ | This lab |
| N∆*zur* | | *zur* deletion mutant of strain NEAU001, Cb^r^ | This lab |
| N∆*rsmA* | | *rsmA* deletion mutant of strain NEAU001, Cb^r^ | This lab |
| N∆*hrpG* | | *hrpG* deletion mutant of strain NEAU001, Cb^r^ | This lab |
| N∆*hrpX* | | *hrpX* deletion mutant of strain NEAU001, Cb^r^ | This lab |
| N∆*pgk* | | *pgk* deletion mutant of strain NEAU001, Cb^r^ | This study |
| CN∆*pgk* | | N∆*pgk* harboring pCpgk, Cb^r^, Sp^r^ | This study |
| N∆*pgk*(*clp*) | | N∆*pgk* harboring pCclp, Cb^r^, Sp^r^ | This study |
| **Plasmids** | |  |  |
| pMD18-T | | pUC *ori*, cloning vector, Ap^r^ | Takara |
| pKMS1 | | Suicide vector derivative from pK18mobGII, *sacB*^+^, Kan^r^ | This lab |
| pHM1 | | Sp^r^ or Sm^r^ *IncW*, *Mob(p), Mob^+^, LacIP^+^*, PK2 replicon, cosmid | This lab |
| pKΔpgk | | A 933 bp fusion cloned in pKMS1 for a 1172 bp deletion in *pgk*, Kan^r^ | This study |
| pCpgk | | pHM1 expressing *pgk* under its own promoter, Sp^r^ | This study |
| pCclp | | pHM1 expressing *clp* under its own promoter, Sp^r^ | This study |

Ap^r^ = ampicillin resistance, Kan^r^ = kanamycin resistance, Sp^r^ = spectinomycin resistance, Cb^r^ = carbenicillin resistance.

**TABLE S2 |** Primers used in this study

| **Purpose** | **Primes** | **Sequence (5’→3’; restriction sites underlined)** | **Description** |
| --- | --- | --- | --- |
| Mutagenesis | *pgk*-1F/  *pgk*-1R | TTCCCGGGTATGCGCTGGTGGCCGGCAGT/  ATGAATTCGGGCTCAGCTCGTTCGGTCTA | A 601 bp fragment left to *pgk* |
|  | *pgk*-2F/  *pgk*-2R | ATGAATTCGTGAGTGCAGCCACGCTGTTT/  TATCTAGATCACCACGGCCAGGCGATGAC | A 322 bp fragment right to *pgk* |
| PCR  verification | *pgk*-1F/  *pgk*-2R | TTCCCGGGTATGCGCTGGTGGCCGGCAGT/  TATCTAGATCACCACGGCCAGGCGATGAC | A 2095 bp fragment including the left and right fragments |
|  | *pgk*-3F/  *pgk*-3R | TGGATGTCGGCGGCAGTCAGG/  GTCGCCGATCATCACGCAGCC | A 2546 bp fragment extending out of the left and right fragments |
| Complementation construction | *pgk*-F/  *pgk*-R | TAAAGCTTACGGCTGACTGCGACCGCTTC/  ATCTGCAGTCACTGACCGCGGGCCTGGAG | A 1500 bp fragment containing the entire *pgk* and its own promoter |
| By-path complementation construction | *clp*-F/  *clp*-R | AGTCGACCACGTCGGTCTGTCCTTATGG/  ATGGTACCCAGCGGGTGCCGTACAGCAC | A 833 bp fragment containing the entire *clp* and its own promoter |
| qRT-PCR | *ihfA-*F/  *ihfA-*R | GCATTGACGAAAGCGGAGATG/  AGTTTTTGGCCAGGGCGGAAC | *ihfA* fragment, 257 bp |
|  | *pgk*-F1/  *pgk*-R1 | TTGAACGAGCCGGATTTGATC/  ATTCGAACACGCCCACCGGAC | *pgk* fragment, 262 bp |
|  | *gumB*-F/  *gumB*-R | TTGGAAAAGATGGTGGCCGAC/  TGCGAAACACGATGACATTGC | *gumB* fragment, 226 bp |
|  | *gumC*-F/  *gumC*-R | AAGTCGCTGGTTGCATACTCG/  CTCTGGATCAACGGACTGCTG | *gumC* fragment, 209 bp |
|  | *gumD*-F/  *gumD*-R | CGCGACAATTACTTCGTGGTG/  CTGAATCGTCGTGCCATGGTC | *gumD* fragment, 228 bp |
|  | *gumE*-F/  *gumE*-R | GCGGCGTGCTTCCTGCTTGGC/  GCGGCAACCAATGAGACGATG | *gumE* fragment, 242 bp |
|  | *gumF*-F/  *gumF*-R | GTACGTGCAACCTCCGCTGTG/  AGCGACACCGGTAGCACGTCC | *gumF* fragment, 192 bp |
|  | *gumG*-F/  *gumG*-R | TCGTGTTGTCGGGCTGGGTTG/  ATACAGGCGTGACCCATTGGC | *gumG* fragment, 230 bp |
|  | *gumH*-F/  *gumH*-R | CAGGACACGCATCAAGGGCTG/  CTTCATGCGCGATGCGTAGGC | *gumH* fragment, 231 bp |
|  | *gumI*-F/  *gumI*-R | GTTTCTTCTCGATGCGCGATG/  GCGCAGCAGGCTACGTTCACG | *gumI* fragment, 227 bp |
|  | *gumJ*-F/  *gumJ*-R | GCTACTGGGCGCTGGTGGTAC/  TTGTACAAGCCCAGCGCGTCC | *gumJ* fragment, 232 bp |
|  | *gumK*-F/  *gumK*-R | CGGTTCTTCTCGCTGCGTTAC/  ATCTGCCTCGCGCATCCAGTC | *gumK* fragment, 240 bp |
|  | *gumL*-F/  *gumL*-R | ACGTCGTGCGCTGTTCTGGTG/  CTTGCCGTTGATACCGCTACC | *gumL* fragment, 211 bp |
|  | *gumM*-F/  *gumM*-R | GCGCTGGATCCTGGACCACAG/  GAGCAGCCGATACATCCATTC | *gumM* fragment, 145 bp |
|  | *xanA*-F/  *xanA*-R | AGCCATCCTTGCCAAACAGCC/  GCGATCAGCAGCCACGGAATC | *xanA* fragment, 240 bp |
|  | *xanB*-F/  *xanB*-R | ATCCGCTATTGCTGGTGCTGC/  GCGGTCGCGAGATCGGGCTTC | *xanB* fragment, 223 bp |

**FIGURE S1**


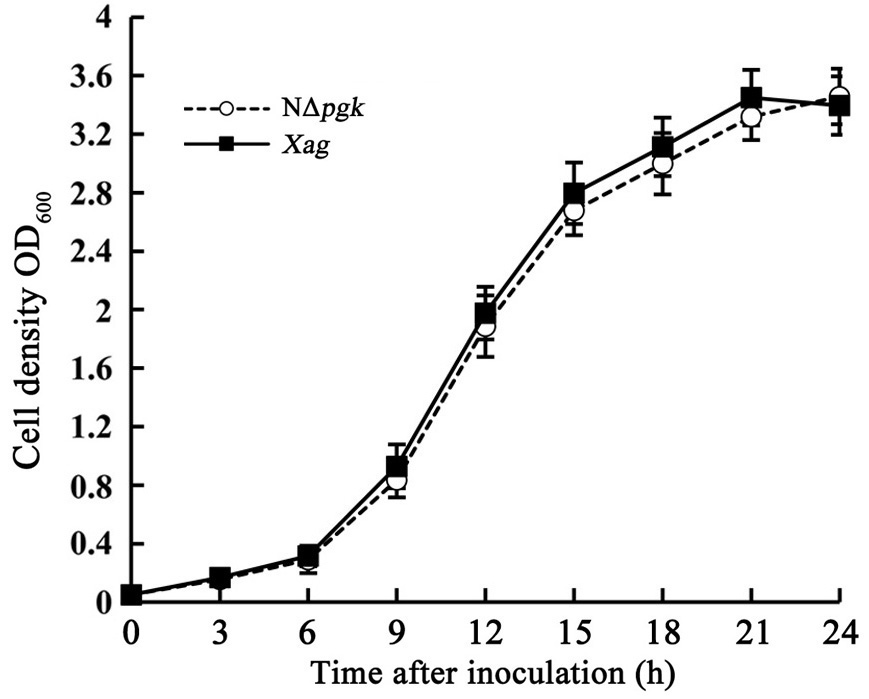


**FIGURE S2**


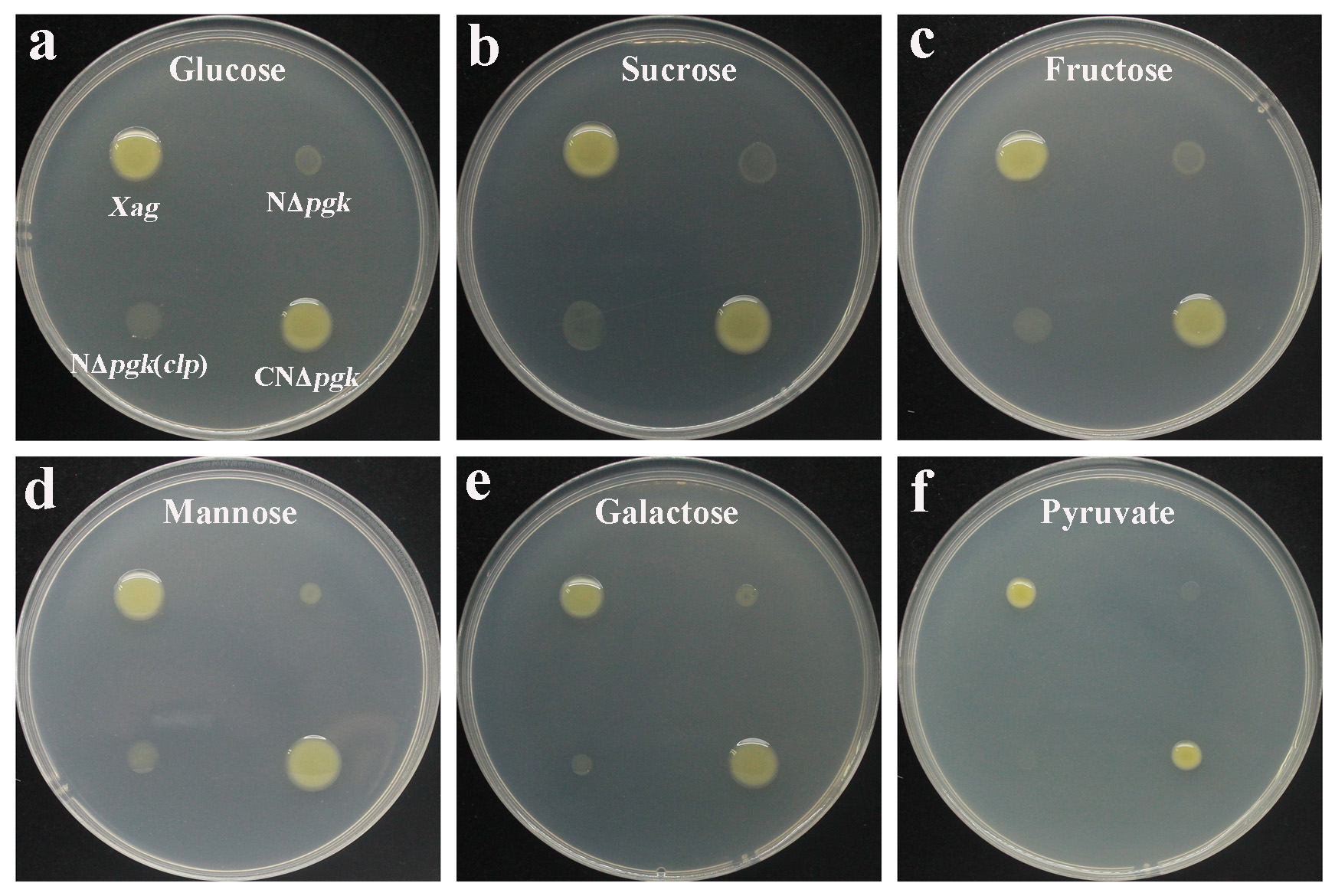


**FIGURE S3**


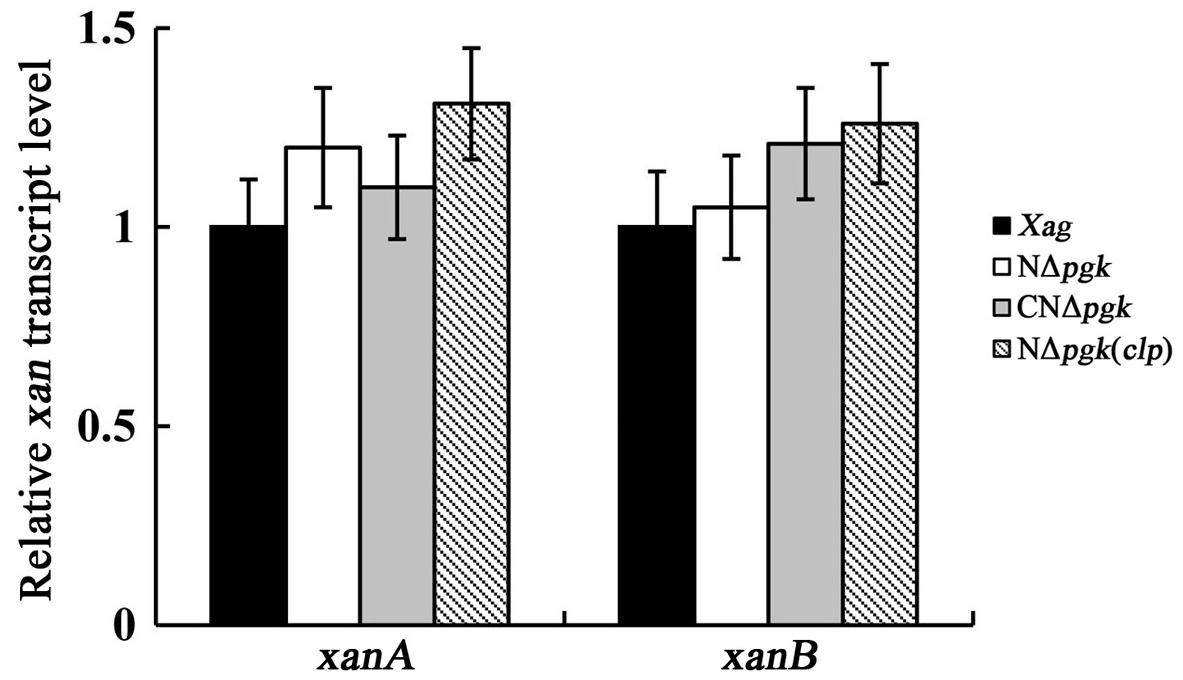


**FIGURE S4**


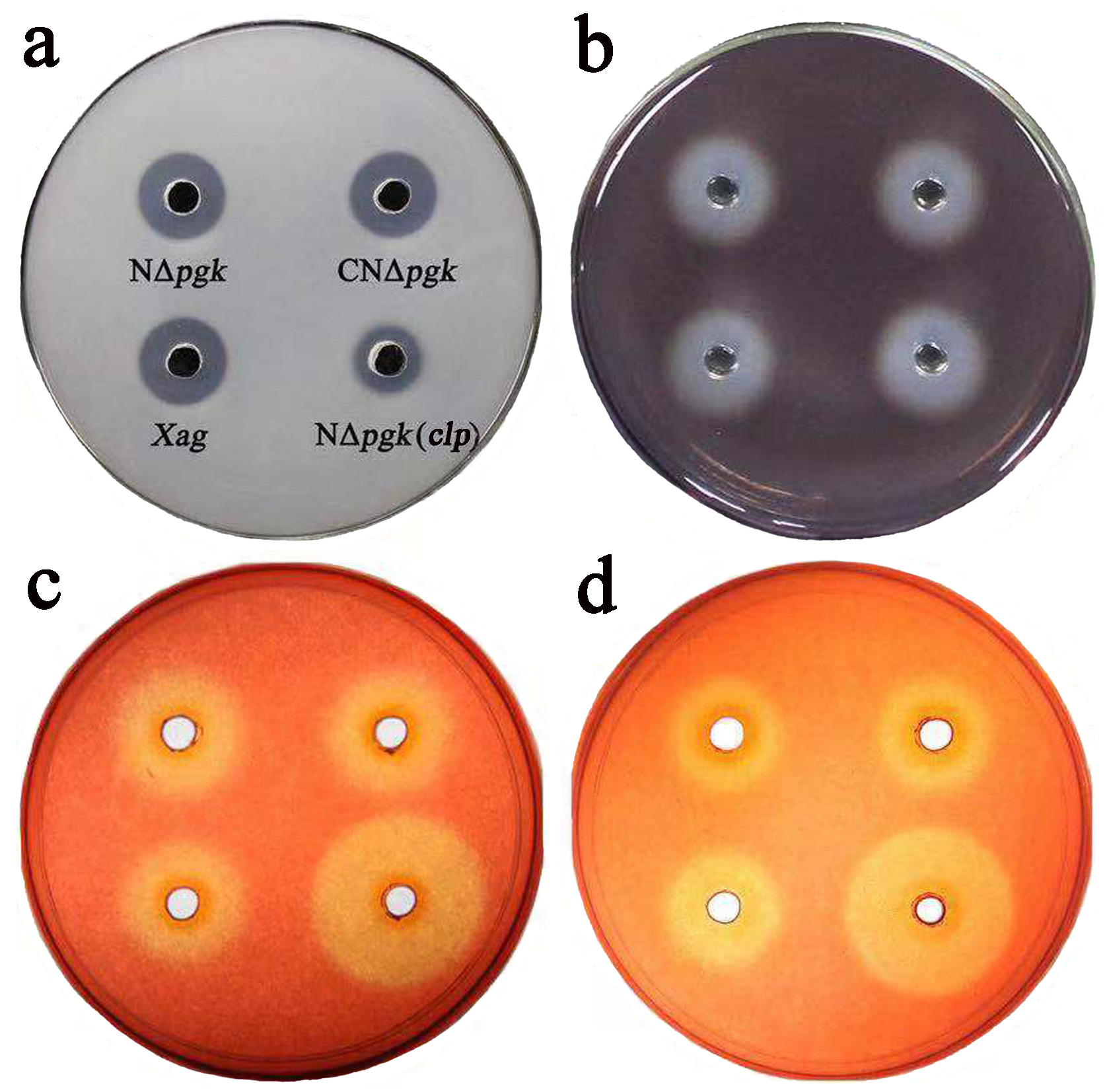


**FIGURE S5**


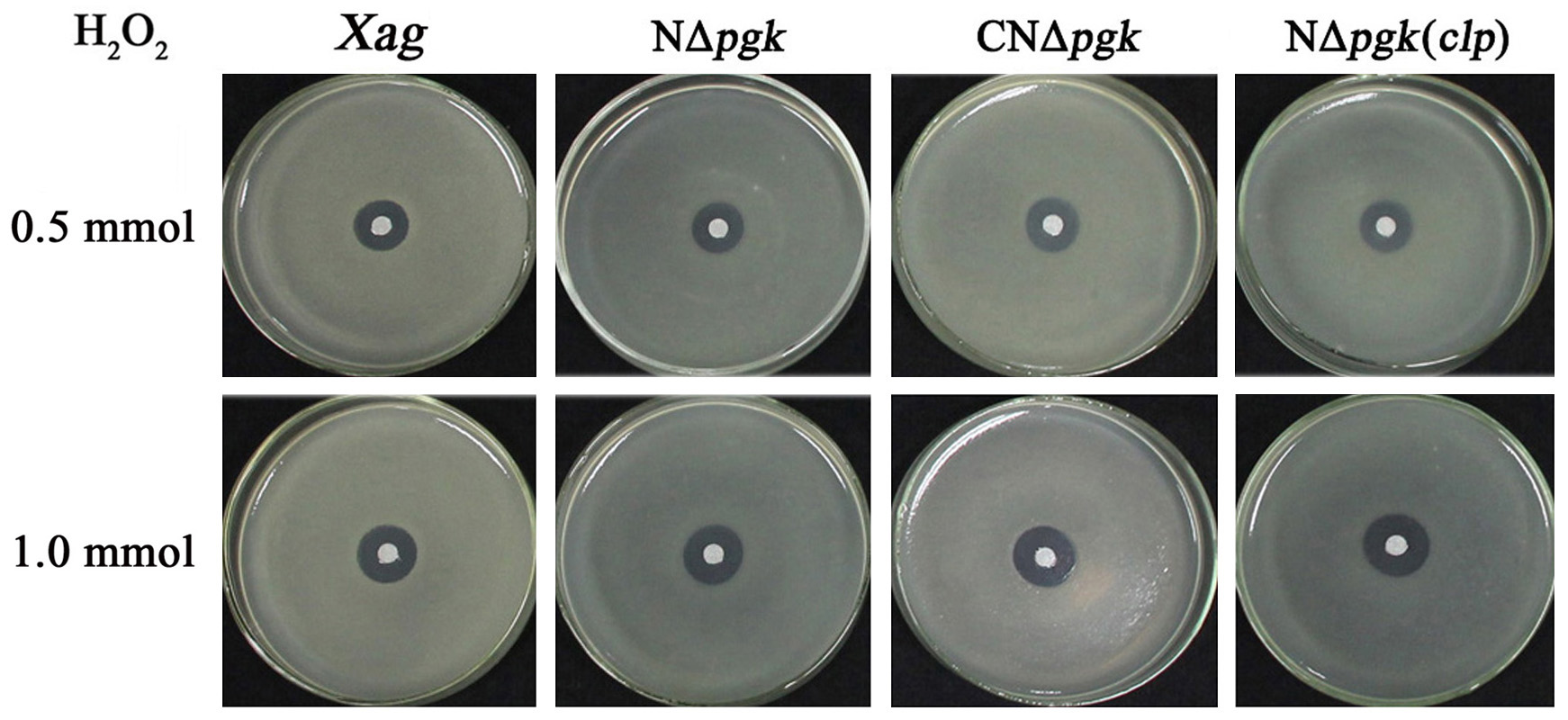

Supplement: Supplementary file 1 [file Data_Sheet_1.docx]
